# Anxiety and Depression in Women’s Cardiovascular Health: Risk Modifiers, Mechanisms and Clinical Implications

**DOI:** 10.3390/jcdd13070301

**Published:** 2026-07-01

**Authors:** Lucio Giuseppe Granata, Simona Giubilato, Clea Giuffrida, Daniela Pavan, Marco Mojoli, Nadia Aspromonte, Isabella Fumarulo, Marcello Marchetta, Adriana Sbrigata, Calogera Pisano, Giuseppina Maura Francese

**Affiliations:** 1 Cardiology Division, Garibaldi-Nesima Hospital, ARNAS Garibaldi, 95122 Catania, Italy; 2 Cardiology Division, Cannizzaro Hospital, 95124 Catania, Italy; simogiub@hotmail.com; 3 Psychologist and Cognitive Behavioral Psychotherapist, 95124 Catania, Italy; cleagiuffrida@gmail.com; 4 Division of Cardiology, Santa Maria degli Angeli Hospital, Azienda Ospedaliera Friuli Occidentale (ASFO), 33170 Pordenone, Italy; daniela.pavan@asfo.sanita.fvg.it (D.P.);; 5 Department of Cardiovascular Sciences, Fondazione Policlinico Universitario A. Gemelli IRCCS, 00168 Rome, Italy; nadia.aspromonte@policlinicogemelli.it (N.A.); isabella.fumarulo@guest.policlinicogemelli.it (I.F.); 6 Department of Cardiovascular Sciences, Catholic University of the Sacred Heart, 00168 Rome, Italy; 7 Department of Life Science, Health, and Health Professions, Link Campus University, 00165 Rome, Italy; 8 Cardiology Department, Policlinico Tor Vergata, 00133 Rome, Italy; marcello.marchetta1997@gmail.com; 9 Cardiac Surgery Unit, Department of Precision Medicine in Medical Surgical and Critical Area (Me.Pre.C.C.), University of Palermo, 90127 Palermo, Italy; adriana.sbrigata@gmail.com (A.S.); calogera.pisano01@unipa.it (C.P.)

**Keywords:** anxiety, depression, women, cardiovascular disease, psychosocial stress, sex differences, gender differences, prevention, takotsubo syndrome, women’s cardiovascular health

## Abstract

Cardiovascular disease is the leading cause of death in women, yet prevention and management have historically relied on male-centered models. Sex and gender critically influence risk, clinical presentation, and outcomes. Depression, anxiety, and psychosocial stress, more prevalent in women, act as key amplifiers of cardiovascular risk. We conducted a clinically oriented narrative review based on a broad, non-systematic search of major databases, integrating evidence selected for relevance and methodological robustness to clarify biological and psychosocial mechanisms linking mental health and cardiovascular disease in women. Affective disorders and stress contribute to cardiovascular risk through interconnected pathways, including hormonal fluctuations, autonomic and neuroendocrine dysregulation, inflammation, endothelial dysfunction, and heightened platelet reactivity. These mechanisms interact with gender-related exposures such as caregiving burden, occupational stress, and interpersonal violence. Stress-related phenotypes, including mental stress, induced ischemia and takotsubo syndrome, exemplify the heart-brain axis and its clinical implications. Incorporating mental health into cardiovascular risk assessment is essential for precision prevention in women. A women-centered approach should include systematic psychosocial evaluation, multidisciplinary care, and tailored strategies to improve risk control, adherence, and outcomes.

## 1. Introduction

Cardiovascular (CV) disease has historically been framed as a predominantly “male” condition and, as a consequence, has often been underestimated in women. In reality, it remains the leading cause of mortality and disability among women worldwide. Contemporary CV medicine increasingly recognizes that women may present with distinct clinical patterns and outcomes across the spectrum of coronary syndromes and prevention, reflecting the interplay of sex-related biology and gender-related exposures [1]. These differences influence symptom expression, diagnostic pathways, healthcare access, therapeutic implementation, and long-term risk-factor control, with persistent gaps in awareness and delivery of guideline-based care [2,3].

Within this framework, sex and gender should be considered complementary determinants of CV risk and care; indeed, relevant differences extend not only to traditional risk factors shared by both sexes, but also to female-predominant or female-specific conditions that may shape cardiovascular vulnerability across the life course. Among these, mental health disorders, particularly depression and anxiety, deserve specific attention. These conditions substantially impair psycho-physical functioning and may adversely influence cardiovascular trajectories and outcomes through behavioral, neuroendocrine, inflammatory, autonomic, and vascular mechanisms. Importantly, the epidemiology, determinants, clinical expression, and cardiovascular consequences of anxiety and depression are themselves influenced by sex-related biological factors and gender-related psychosocial exposures; therefore, understanding the relationship between mental health and cardiovascular disease in women requires a sex- and gender-informed framework that integrates biological susceptibility with psychosocial context across the life course [2,3,4]. Incorporating psychosocial determinants into women-focused CV prevention is therefore not optional, but integral to a comprehensive, precision-oriented risk appraisal [4].

**Figure 1:**
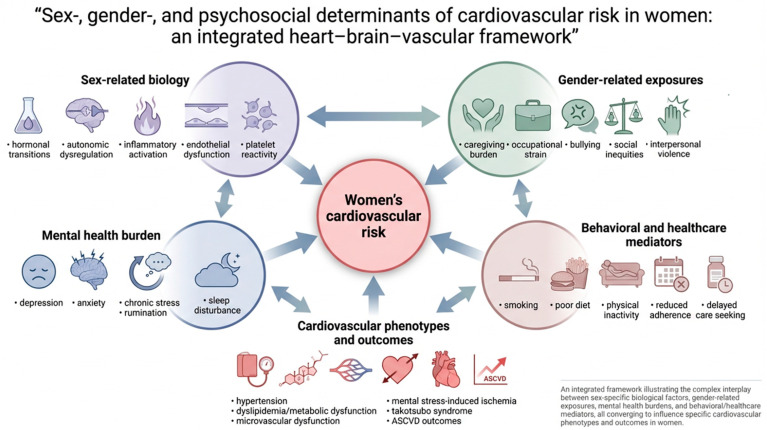
Sex-, gender-, and psychosocial determinants of cardiovascular risk in women: an integrated heart–brain–vascular framework. This figure illustrates the multidimensional pathways through which sex-related biological factors, gender-related exposures, psychosocial burden, and behavioral and healthcare mediators interact to shape cardiovascular risk in women. Hormonal transitions, autonomic and neuroendocrine dysregulation, inflammatory activation, endothelial dysfunction, platelet reactivity, caregiving burden, occupational strain, bullying, social inequities, and interpersonal violence may converge with depression, anxiety, chronic stress, rumination, and sleep disturbance to amplify cardiovascular vulnerability. These mechanisms may promote maladaptive health behaviors, impaired adherence, delayed care seeking, cardiometabolic dysfunction, microvascular ischemia, takotsubo syndrome, and adverse atherosclerotic cardiovascular outcomes. The figure emphasizes the need for a sex-informed and bio-psycho-social model of cardiovascular prevention in women and is intended as a conceptual framework that integrates the principal domains discussed in this review, illustrating their potential interactions rather than providing an exhaustive representation of each individual mechanism or psychosocial determinant.

In this context, understanding the cardiovascular implications of mental health in women requires consideration not only of anxiety and depression, but also of broader psychosocial determinants, including chronic stress, interpersonal violence, female-specific vulnerability windows, and stress-related cardiovascular phenotypes. This review provides a sex- and gender-informed overview of these interconnected domains, with particular emphasis on the biological and behavioral mechanisms linking mental health to cardiovascular risk, the emerging heart–brain–vascular framework, and the clinical implications for prevention and multidisciplinary care. The multidimensional interplay between sex-related biology, gender-related exposures, psychosocial burden, and CV vulnerability in women is summarized in Figure 1.

## 2. Materials and Methods

This narrative review was designed to provide a clinically oriented synthesis of the relationship between anxiety, depression, psychosocial stress, and cardiovascular disease in women, integrating current evidence on sex-related biological mechanisms and gender-related determinants of cardiovascular vulnerability.

A broad literature search was performed in PubMed, Scopus and Embase to identify relevant studies published up to January 2026. The search strategy combined free-text terms related to mental health and psychosocial burden, including “anxiety”, “depression”, “psychological stress”, “psychosocial factors”, “violence”, and “intimate partner violence”, with terms related to CV disease and women’s CV health, including “cardiovascular disease”, “coronary artery disease”, “acute coronary syndromes”, “myocardial ischemia”, “microvascular dysfunction”, “takotsubo syndrome”, “women”, “female sex”, “sex differences” and “gender differences”.

The electronic search identified several hundred potentially relevant records. After removal of duplicate records and screening of titles and abstracts, full-text articles were assessed for thematic relevance. Two reviewers independently screened titles, abstracts, and full texts for eligibility. Any disagreements regarding study inclusion were resolved through discussion and consensus. The final narrative synthesis incorporated evidence from observational studies, cohort studies, systematic reviews, meta-analyses, narrative reviews, guideline documents, consensus statements, and selected mechanistic investigations. To ensure comprehensive coverage of the topic, the electronic search was supplemented by hand-searching the reference lists of key articles, relevant reviews, position papers, and perspective documents to identify additional studies of potential relevance. Additional articles were included when considered important to contextualize mechanistic pathways, sex- and gender-related determinants, female-specific vulnerability windows, and prevention-oriented clinical implications. The review prioritized studies of greater clinical and translational relevance, including observational studies, cohort studies, meta-analyses, systematic reviews, narrative reviews, guideline documents, and selected mechanistic investigations. Particular emphasis was placed on literature addressing the heart–brain–vascular interplay, stress-related CV phenotypes, and the impact of anxiety and depressive disorders on CV risk, presentation, and long-term outcomes in women. Given the narrative nature of the manuscript, no formal predefined inclusion or exclusion criteria were applied, and no structured quality assessment was performed. The final selection of the literature was guided by thematic relevance, methodological credibility, recency of evidence, and the contribution of each study to a coherent and up-to-date synthesis of the field. The conduct and reporting of this narrative review were informed by established recommendations for high-quality narrative reviews, with particular attention to transparency of literature identification, source selection, and thematic synthesis.

## 3. Depression, Stress, and Female Cardiovascular Risk

### 3.1. Clinical and Psychosocial Burden of Depression in Women

Depression represents one of the most prevalent and clinically relevant mental health conditions affecting women and is increasingly recognized as an important modifier of cardiovascular risk and prognosis [5]. Multiple studies report a higher prevalence of depression in women and show that depression is associated with worse cardiovascular prognosis, with some analyses suggesting a stronger association in women than in men [6,7,8]. In women, major depression (the most severe form) appears approximately twice as often as in men, while dysthymia is reported up to three times more frequently [9,10]; in fact, sex-related differences have been described in age of onset, clinical course, symptom profile, and response to treatment, including pharmacological treatment (with higher antidepressant use) and psychotherapy [11,12,13].

The biological and psychosocial reasons behind these differences are actively debated because women appear to experience a higher exposure to vulnerability windows across the life course, including female-specific phases in which depressive symptoms may emerge or worsen, such as menarche and cyclical hormonal fluctuations (including premenstrual dysphoric disorder, reported in approximately 1.5–2% of women) and the postpartum period (with postpartum depression affecting up to ~12% of new mothers) [14,15,16]. Beyond biological vulnerability windows, psychosocial stressors play a major role as highlighted by potentially destabilizing life events, including bereavement, unemployment, financial strain, family dysfunction or conflict, and exposure to violence, that are consistently associated with a higher depressive burden in women [4,17,18,19,20,21]. Sociocultural determinants, including role strain and work–family conflict, may further amplify this vulnerability [22,23], and more speculatively, persistent self-doubt related to role performance, sometimes conceptualized as the impostor phenomenon, may represent an additional contributor to psychological distress in some women [24,25]. Mental health disorders have also been linked to adverse workplace dynamics, including bullying and power imbalance, which may contribute to chronic psychological distress and sickness absence [26,27]. From childhood onward, gendered socialization may shape emotion regulation and coping styles; notably, across many sociocultural contexts, boys are more often encouraged toward autonomy, assertiveness, and risk-taking, whereas girls may receive stronger reinforcement for self-control, relational sensitivity, and inward-oriented regulation [28,29]. These cultural, social, and educational influences may contribute to a greater tendency among women to internalize distress, including through rumination, repetitive negative thinking, and self-critical processing [30,31,32,33]. Women are more frequently exposed to abuse and physical risk and sexual harassment, domestic violence, and sexual abuse are disproportionately reported, with wide prevalence estimates (15–71%) [34,35]. Epidemiological studies have also documented higher risk of chronic medical conditions among individuals exposed to intimate partner violence [35,36,37], and traumatic experiences, in turn, increase vulnerability to depression [20]. Even in the absence of overt violence, women appear more sensitive to the depressogenic effects of interpersonal problems within close social networks [38]. Moreover, perceived stress and exposure to adverse life events have been associated with increased cardiovascular vulnerability in women, particularly at younger and midlife ages [39,40].

Compared with previous decades, psychosocial stress may have increased in women alongside growing participation in economic, social, political, and occupational roles. Many women carry multiple social roles during midlife, increasing cumulative burden and, importantly, the adverse impact of role load may be modulated by the degree to which these roles are experienced as rewarding [41,42]. Individual differences in stress reactivity have been proposed as an important contributor to sex-related health differences [43,44], with some evidences suggesting that women may be more likely to respond to stress through rumination and self-focused coping, whereas men may more often employ distraction strategies. However, these patterns are influenced by cultural, social, and individual factors and should not be considered universal [45]. In men, distress may also manifest more outwardly, with action-oriented responses such as irritability, ideational acceleration, disinhibition, and a higher tendency toward poorly controlled behaviors and impulsivity, potentially linked to reduced insight, with action serving as an attempt to attenuate suffering [46]; conversely, women more frequently show inward-directed emotional processing, with prominent worry, fear, anguish, reduced interest and energy, and diminished self-confidence [30,47,48]. The COVID-19 pandemic was also associated with increased depression and stress, particularly among women; the traumatic experience of quarantine was linked to higher depressive symptoms, stress, and insomnia, fostering unhealthy lifestyle patterns (unhealthy diet and reduced physical activity) [49,50,51,52,53]. Beyond epidemiological and psychosocial determinants, depression in women is increasingly recognized as a biologically active condition, capable of modulating CV risk through multiple interconnected pathways [54].

### 3.2. Biological Pathways Linking Depression to Cardiovascular Risk in Women

Depression in women is associated with a complex interplay of neuroendocrine, inflammatory, autonomic, and vascular mechanisms that collectively contribute to increased CV risk. These pathways extend beyond behavioral factors and support the concept of depression as a biologically active condition capable of modulating CV physiology [55]. A plausible hypothesis is that women may develop sustained activation of inflammatory pathways that becomes chronic over time [56,57], and it may be accompanied by suboptimal regulation of the hypothalamic–pituitary axis and the serotonin–kynurenine pathway, reinforcing a pro-inflammatory state that promotes endothelial dysfunction and platelet activation in a self-perpetuating vicious circle [54,58,59]. In parallel, dysregulation of the autonomic nervous system with sympathetic predominance may contribute [60,61]. A central mechanism involves dysregulation of the hypothalamic–pituitary–adrenal (HPA) axis with chronic activation of stress-related neuroendocrine pathways leading to sustained cortisol exposure, which may promote metabolic disturbances, visceral adiposity, insulin resistance, and endothelial dysfunction [54,56]. Altered HPA axis responsiveness has been described across the female reproductive lifespan, suggesting that hormonal transitions may further amplify vulnerability to stress-related CV effects in women [56,62], with inflammatory activation representing another key pathway linking depression to CV disease. Indeed, women with depressive symptoms have been shown to exhibit elevated levels of pro-inflammatory cytokines, including interleukin-6 and tumor necrosis factor-α, as well as increased C-reactive protein levels, thus chronic low-grade inflammation contributes to endothelial dysfunction, oxidative stress, and progression of atherosclerosis [54]. Notably, early-life and prenatal exposure to depressive states may also influence long-term inflammatory profiles, further reinforcing CV vulnerability [57].

Depression is also associated with abnormalities in platelet function and vascular reactivity; enhanced platelet activation and aggregation have been described in depressed individuals, potentially mediated by alterations in serotonin signaling and the tryptophan-kynurenine pathway [58,59], mechanisms that may increase thrombotic risk and contribute to adverse CV outcomes, particularly in the setting of acute coronary syndromes [63]. Autonomic nervous system imbalance further contributes to the CV burden of depression; in fact, reduced heart rate variability and a shift toward sympathetic predominance have been consistently reported, reflecting impaired vagal tone and altered CV regulation. This autonomic dysregulation may increase susceptibility to arrhythmias, impair myocardial perfusion, and lower the threshold for ischemic events [60,61].

Importantly, these biological pathways do not operate in isolation but rather converge within an integrated heart–brain–vascular network. Mental stress and depressive symptoms can trigger dynamic hemodynamic and coronary microvascular responses, with accumulating evidence indicating sex-specific patterns: women appear more likely to develop stress-induced myocardial ischemia mediated by microvascular dysfunction rather than epicardial coronary obstruction [61,64]. This pathophysiological framework offers a coherent mechanistic link between affective disorders and the higher prevalence of ischemia with non-obstructive coronary arteries (INOCA) observed in women [65]. Beyond their contribution to long-term atherosclerotic risk, psychosocial stressors thus act as acute modulators of ischemic burden, shaping clinically relevant symptom trajectories through sex-dependent differences in vascular reactivity and myocardial perfusion [66,67,68,69].

Overall, the convergence of neuroendocrine dysregulation, inflammation, autonomic imbalance, and platelet activation supports the interpretation of depression as a multidimensional CV risk modifier in women, operating through both systemic and vascular-specific pathways [55,61].

### 3.3. Behavioral and Adherence-Related Pathways

Beyond biological mechanisms, depression in women influences CV risk through a range of behavioral and healthcare-related pathways that may substantially affect disease progression and outcomes. These pathways are particularly relevant in clinical practice, as they represent modifiable targets for intervention [55].

Depressive symptoms are consistently associated with adverse health behaviors, including reduced physical activity, unhealthy dietary patterns, smoking, and, in some cases, increased alcohol or substance use, contributing to the development and progression of cardiometabolic risk factors such as obesity, hypertension, dyslipidemia, and impaired glucose metabolism [4,6,70]. In women, the impact of these behaviors may be further amplified by psychosocial stressors and competing life demands, including caregiving responsibilities and occupational burden [4].

Depression is also linked to reduced engagement with healthcare systems and lower adherence to prescribed therapies. Women with depressive symptoms may be less likely to attend follow-up visits, participate in preventive programs or cardiac rehabilitation, and maintain long-term adherence to pharmacological treatments, including lipid-lowering, antihypertensive, and antiplatelet therapies [7,71]. This reduced adherence contributes to suboptimal control of key CV risk factors and may partially explain worse outcomes observed in patients with coexisting depression and CV disease [63]; in addition, depression may influence symptom perception and healthcare-seeking behavior. Women are more likely to present with atypical or less specific CV symptoms, and the presence of depressive or anxiety symptoms may further delay recognition and timely access to care [3]. This delay can be particularly relevant in acute settings, where early diagnosis and treatment are critical for improving outcomes.

Importantly, these behavioral and healthcare-related mechanisms often interact with underlying biological processes, creating a self-reinforcing cycle in which depression promotes adverse behaviors and reduced adherence, which in turn exacerbate cardiometabolic risk and disease progression [72,73]. Recognizing and addressing these pathways is therefore essential for translating the concept of depression as a CV risk modifier into actionable, patient-centered prevention strategies in women [74,75].

## 4. Anxiety and Sex-Related Vulnerability

### 4.1. Clinical and Neuropsychological Features of Anxiety in Women

Anxiety disorders are highly prevalent in women, with epidemiological data consistently showing approximately a twofold higher prevalence compared with men [47,76]. These differences emerge early in life and persist across the lifespan, suggesting the contribution of both biological and psychosocial determinants [45,47]. Clinically, anxiety disorders encompass a spectrum of conditions characterized by excessive fear, anticipatory worry, heightened arousal, and avoidance behaviors [77,78]. A useful conceptual distinction is that between anxious apprehension, a future-oriented cognitive state dominated by worry, and fear, a more immediate response to perceived threat [79,80]; women are more likely to exhibit patterns of anxious apprehension, with prominent rumination, hypervigilance, and internalized distress, which may contribute to the persistence and chronicity of symptoms [30,47].

Sex-related differences in anxiety also extend to symptom expression and comorbidity patterns. Women more frequently present with generalized anxiety disorder, panic disorder, and specific phobias, often in association with depressive symptoms [47]. These conditions are associated with significant functional impairment, including sleep disturbances, reduced quality of life, and impaired occupational and social functioning [77]. Importantly, anxiety in women often shows a temporal relationship with hormonal fluctuations across the reproductive lifespan. Increased vulnerability has been described during puberty, the premenstrual phase, pregnancy and postpartum, and the menopausal transition, supporting the role of hormonal modulation in shaping anxiety-related phenotypes [81]. Neurobiological mechanisms underlying these patterns include sex-specific modulation of limbic circuits involved in emotional processing, as well as differences in serotonergic signaling and stress responsivity [82].

### 4.2. Biological and Cardiovascular Correlates of Anxiety

Beyond its psychological and behavioral manifestations, anxiety is associated with a range of biological alterations that may influence CV function and contribute to disease risk. Central to this relationship is the activation of stress-responsive neurobiological systems, including the autonomic nervous system and neuroendocrine pathways [83]. Similar to depression, anxiety disorders in women often precipitate or worsen during periods of hormonal fluctuation, including puberty, the premenstrual phase, pregnancy or postpartum, and the menopausal transition [81]. Female susceptibility to anxiety appears driven not only by hormonal and biological influences but also by sex-related differences in stress response [84], with preliminary evidence suggesting that sex-related differences in oxytocin signaling and stress responsivity may contribute to anxiety responses, although the underlying mechanisms remain incompletely understood and require further investigation [85]. Neurobiological correlates have also been proposed, including potential sex-related differences in limbic structure and function, although available findings remain heterogeneous and should not be interpreted as definitive markers of anxiety vulnerability [82]. Several studies have suggested that serotonergic signaling may act as a sex-sensitive psychobiological interface. Women may exhibit sex-related differences in serotonin regulation, a key neurotransmitter involved in emotional behavior, potentially influenced by hormonal fluctuations across the reproductive lifespan [81]. Genetic contributors have also been discussed, including variants within the serotonin transporter gene-linked polymorphic region (5-HTTLPR/SLC6A4), which has been implicated in serotonin regulation and stress-related affective vulnerability, although its effects appear context-dependent and should not be interpreted deterministically [86,87]. In addition, sex-related differences in brain structure and function have been described in cortico-limbic circuits involved in emotional processing and regulation, including the prefrontal cortex, amygdala, and broader limbic system [88,89,90]. These differences, together with hormonal modulation of neural circuits, have been hypothesized to contribute to sex-specific patterns of anxiety and affective disorders, although causal relationships remain to be fully established [81,82].

**Figure 2:**
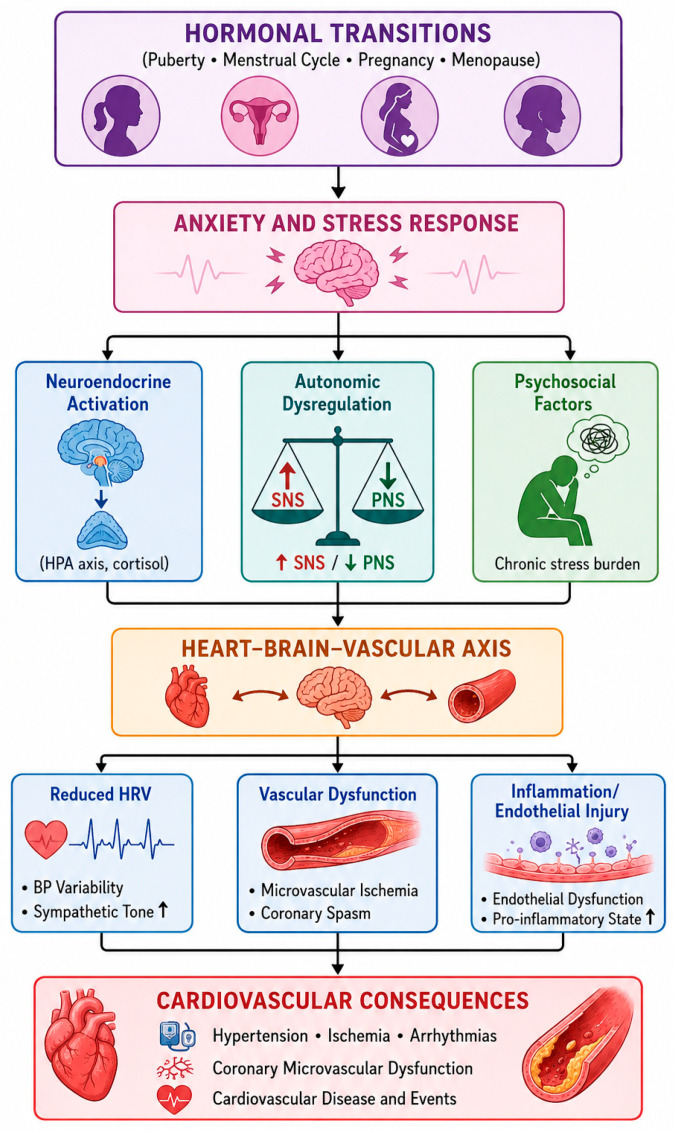
The heart–brain–vascular axis linking anxiety to cardiovascular risk in women. The figure illustrates how hormonal transitions across the female lifespan may interact with anxiety-related stress responses, neuroendocrine activation, autonomic dysregulation, and psychosocial burden. These pathways converge on the heart–brain–vascular axis, contributing to impaired cardiovascular regulation, vascular dysfunction, inflammatory activation, microvascular ischemia, arrhythmias, and adverse CV outcomes. The model highlights the multidirectional relationship between psychological health and cardiovascular physiology in women. Abbreviations: BP, blood pressure; CV, cardiovascular; HPA, hypothalamic–pituitary–adrenal; HRV, heart rate variability; PNS, parasympathetic nervous system; SNS, sympathetic nervous system.

To facilitate understanding of the complex interactions between anxiety, psychosocial stress, and cardiovascular vulnerability in women, Figure 2 provides an integrated conceptual representation of the heart–brain–vascular axis. The model summarizes the principal biological, autonomic, neuroendocrine, and psychosocial pathways through which anxiety may influence cardiovascular regulation and contribute to adverse cardiovascular phenotypes across the female life course.

Anxiety states are characterized by increased sympathetic activity and reduced parasympathetic tone, resulting in autonomic imbalance and impaired CV regulation. As discussed above for depression, these pathways may contribute to reduced heart rate variability, heightened CV reactivity, arrhythmogenic vulnerability, and impaired coronary perfusion [61]. Consistent with the heart–brain–vascular paradigm, anxiety-related physiological responses may also contribute to stress-mediated myocardial ischemia through hemodynamic and microvascular pathways, particularly in women and even in the absence of significant epicardial coronary obstruction [64,66,68]. Overall, anxiety in women should be interpreted as a multidimensional condition with neuropsychological, biological, and CV correlates. Its integration into CV risk assessment frameworks may help identify vulnerable individuals and support more comprehensive, sex-informed prevention strategies [62,83,91].

Importantly, the biological and CV impact of anxiety in women is not static but evolves across the life course, reflecting the dynamic interplay between hormonal transitions, psychosocial exposures, and neuroendocrine regulation.

The principal biological and behavioral pathways through which anxiety and depression may influence CV risk in women are summarized in Table 1.

## 5. Female-Specific Vulnerability Windows Across the Life Course

Women’s vulnerability to anxiety, depression, and stress-related cardiovascular consequences is not uniformly distributed across the lifespan but clusters around specific hormonal and reproductive transitions. These phases represent periods of heightened biological and psychosocial susceptibility, during which the neuroendocrine, autonomic, inflammatory, and vascular pathways discussed above may amplify both affective symptom burden and CV risk [14,56,81].

**Figure 3:**
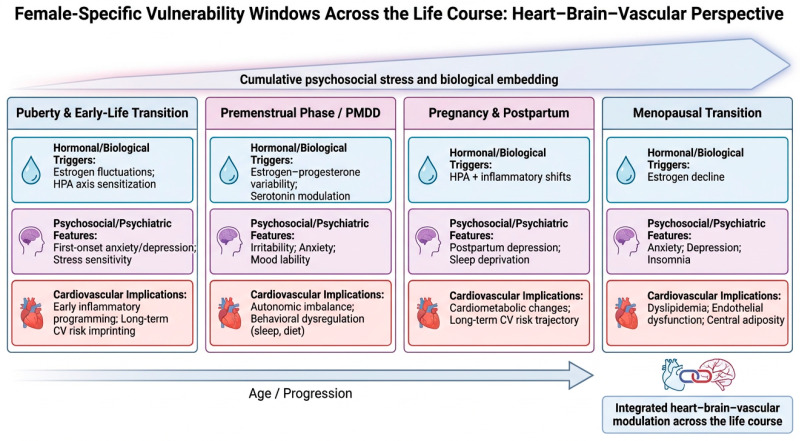
Female-specific life-course windows of affective and cardiovascular vulnerability. This figure summarizes the main female-specific windows during which hormonal transitions, psychosocial stressors, and neuroendocrine changes may amplify vulnerability to anxiety, depression, and cardiovascular dysfunction. Across puberty/adolescence, the premenstrual phase, pregnancy/postpartum, and the menopausal transition, biological and psychosocial determinants interact to influence emotional regulation, autonomic balance, inflammation, endothelial function, and cardiometabolic risk. The scheme highlights these stages as critical opportunities for tailored screening, risk stratification, and integrated preventive interventions. Abbreviation: CV, cardiovascular; HPA, hypothalamic–pituitary–adrenal; PMDD, Premenstrual dysphoric disorder.

Female-specific hormonal and reproductive transitions create distinct windows of affective and cardiovascular vulnerability across the life course, as outlined in Figure 3.

### 5.1. Early-Life Transition: Puberty and Reproductive Onset

Puberty represents one of the earliest divergence points in sex differences in mental health. The incidence of both depressive and anxiety disorders increases markedly in girls during adolescence, coinciding with the onset of gonadal hormonal activity and maturation of stress-responsive neurocircuitry [45,81]. Fluctuations in estrogen and progesterone concentrations across the reproductive lifespan have been shown to influence serotonergic neurotransmission and neural circuits involved in emotional processing and stress regulation, potentially contributing to sex-specific patterns of anxiety vulnerability and symptom expression [88,92,93]. In parallel, early-life exposure to psychosocial stressors, including family instability, social pressures, and adverse experiences, may exert long-term effects on neuroendocrine and inflammatory pathways. Evidence suggests that early exposure to stress and depressive states may influence inflammatory programming and CV risk trajectories later in life [57]. These findings support the concept that adolescence is not only a psychiatric vulnerability period but also a formative phase for long-term CV risk modulation in women.

### 5.2. Cyclical Hormonal Vulnerability: Premenstrual Phase and Premenstrual Dysphoric Disorder

The premenstrual phase represents a recurring window of vulnerability characterized by cyclical hormonal fluctuations that may precipitate mood and anxiety symptoms in susceptible individuals [81]. Premenstrual dysphoric disorder (PMDD), affecting a subset of women, is associated with significant emotional, cognitive, and somatic symptoms, including irritability, anxiety, and depressive mood [15]. These cyclical changes are thought to reflect increased sensitivity to normal hormonal fluctuations rather than absolute hormone levels, with downstream effects on neurotransmitter systems, including serotonin pathways [81]. Recurrent affective symptoms during the premenstrual phase may contribute to cumulative stress burden and behavioral dysregulation, potentially influencing CV risk indirectly through adverse health behaviors and autonomic imbalance [5].

### 5.3. Pregnancy and Postpartum: A Cardio-Obstetric Interface

Pregnancy and the postpartum period constitute a complex physiological and psychological transition characterized by profound hormonal, metabolic, and CV changes. Postpartum depression affects a substantial proportion of women and represents one of the most clinically relevant affective conditions in this life stage [16]. Neuroendocrine adaptations during pregnancy, including changes in HPA axis regulation and inflammatory signaling, may predispose vulnerable individuals to mood disturbances [56]. In addition, psychosocial stressors such as caregiving demands, sleep deprivation, and role transition may further increase susceptibility to anxiety and depression [14]. Importantly, these affective conditions may have CV implications. Pregnancy-related complications and postpartum cardiometabolic changes may interact with psychological stress and depressive symptoms, contributing to long-term CV risk. This period therefore represents a key opportunity for integrated screening and early preventive interventions targeting both mental health and CV risk factors [94,95,96].

### 5.4. Menopausal Transition: Convergence of Affective and Cardiometabolic Risk

The menopausal transition is another critical window characterized by hormonal variability, particularly declining estrogen levels, which may influence both mood regulation and CV physiology. Perimenopause has been associated with increased prevalence of depressive and anxiety symptoms, sleep disturbances, and vasomotor symptoms, all of which may interact to amplify overall disease burden [81]. Hormonal fluctuations during this phase may affect central neurotransmitter systems and stress responsivity, contributing to affective vulnerability [43]; at the same time, menopause is associated with adverse changes in CV risk profiles, including increased central adiposity, dyslipidemia, and endothelial dysfunction [94]. The coexistence of affective symptoms and emerging cardiometabolic risk factors during this transition underscores the importance of a comprehensive, women-centered approach to risk assessment. Targeted screening and intervention during the menopausal transition may therefore provide an opportunity to mitigate both psychological distress and CV risk progression [5,75].

## 6. Interpersonal Violence, Trauma, and Chronic Psychosocial Stress

Interpersonal violence and chronic psychosocial stress represent major, yet often underrecognized, determinants of CV risk in women. These exposures are highly prevalent and disproportionately affect women across the lifespan, contributing to both the onset and progression of affective disorders and CV disease [20,97].

Large epidemiological studies have documented substantial rates of intimate partner violence and sexual abuse among women worldwide, with wide variability across populations but consistently high lifetime prevalence [34,36]. Exposure to violence is strongly associated with increased risk of depression, anxiety, and post-traumatic stress disorder (PTSD), conditions that frequently coexist and may exert cumulative effects on CV health [20].

Beyond psychological consequences, interpersonal violence has been linked to adverse cardiometabolic profiles. Cohort studies have demonstrated associations between exposure to partner violence and increased abdominal adiposity, dyslipidemia (lower HDL cholesterol and higher triglycerides), and other metabolic disturbances [37]. Moreover, women with a history of domestic abuse exhibit a significantly higher risk of incident CV disease and ischemic heart disease, suggesting that violence-related stress may have long-term systemic effects [98]. The biological mechanisms underlying these associations are multifactorial and involve sustained activation of stress-response systems. Chronic exposure to trauma may lead to persistent dysregulation of the HPA axis, with metabolic and inflammatory consequences [62,99]. In parallel, chronic stress has been associated with immune activation, endothelial dysfunction, and epigenetic modifications, including telomere shortening, which may accelerate biological aging and vascular damage [97].

Importantly, trauma-related stress may also influence CV risk through behavioral pathways. Women exposed to interpersonal violence are more likely to engage in maladaptive coping strategies, including smoking, unhealthy dietary patterns, physical inactivity, and substance use, all of which contribute to cardiometabolic risk and had a 31% higher risk of developing CV disease, including an approximately 50% increase in ischemic heart disease risk [36,98]. In addition, these individuals may experience barriers to accessing healthcare, reduced trust in medical systems, and lower adherence to preventive and therapeutic interventions [36]. Chronic psychosocial stress extends beyond overt violence and includes persistent exposure to adverse social environments, such as workplace bullying, caregiving burden, financial strain, and social isolation [18,26,42]. Workplace-related stressors, including bullying and power imbalance, have been associated with increased risk of mental health disorders and sickness absence, further contributing to long-term health deterioration [26,27]. These stressors often interact with gendered social roles, amplifying cumulative stress burden in women.

From a life-course perspective, early-life trauma, including childhood physical or sexual abuse, has been associated with an increased risk of early-onset CV events in adulthood, supporting the concept of long-term biological embedding of stress [40]. This cumulative exposure model suggests that repeated or sustained psychosocial stress may progressively impair neuroendocrine regulation, vascular function, and behavioral health, ultimately increasing CV vulnerability [68].

Finally, the COVID-19 era provides a contemporary lens on how population-level stress can amplify CV vulnerability. The psychological impact of quarantine, including heightened stress, anxiety, and depressive symptoms, was widely documented [53], and was paralleled by adverse lifestyle shifts (dietary changes and reduced physical activity) that plausibly increase CV risk [49]. Together with evidence linking workplace bullying and sickness absence and conceptual models clarifying bullying as a chronic psychosocial exposure, these data reinforce the need to incorporate psychosocial determinants into women-centered CV prevention and long-term follow-up [26,27]. Overall, interpersonal violence and chronic psychosocial stress should be recognized as clinically relevant CV risk modifiers in women. Their identification requires a proactive, trauma-informed approach to clinical assessment, with integration of psychosocial history into CV risk evaluation and the development of multidisciplinary care pathways aimed at mitigating both psychological and CV consequences [5].

## 7. The Heart–Brain–Vascular Axis in Women

### 7.1. General Overview

The relationship between anxiety, depression, and CV disease in women can be conceptualized within an integrated heart–brain–vascular axis, in which central nervous system processes, neuroendocrine responses, vascular function, and behavioral patterns interact dynamically to shape cardiovascular risk. Rather than introducing entirely separate mechanisms, this framework integrates the biological and behavioral pathways described above into a systems-level model of disease vulnerability in women [54,100]. At the core of this axis lies the bidirectional communication between the brain and the cardiovascular system. Emotional processing, stress perception, and affective regulation are mediated by interconnected neural circuits involving the amygdala, prefrontal cortex, hippocampus, and hypothalamus, which modulate autonomic and neuroendocrine outputs that directly influence cardiovascular function [61,82,100]. Sex-related differences in these circuits, including structural and functional variations and differential hormonal modulation, may contribute to the heightened susceptibility of women to stress-related cardiovascular effects [81,82]. As detailed in the previous sections, persistent psychological stress and depressive or anxiety states may influence cortisol exposure, sympathetic–parasympathetic balance, immune activation, endothelial function, and thrombotic potential [54,56,58,59,60,61]. In women, these mechanisms may be further shaped by hormonal fluctuations across the reproductive lifespan and by gender-related psychosocial exposures [62]. Rather than acting in isolation, these pathways likely interact in a self-reinforcing network that links affective distress to metabolic dysregulation, endothelial dysfunction, arrhythmogenic susceptibility, atherosclerotic progression, and microvascular dysfunction [55].

Importantly, the heart–brain–vascular axis also encompasses functional and microvascular aspects of myocardial ischemia. Experimental studies have demonstrated that mental stress can induce myocardial ischemia through mechanisms that differ from those associated with physical stress, involving microvascular dysfunction, altered vasomotor tone, and hemodynamic changes [64]. These responses appear to exhibit sex-specific patterns, with women more likely to develop ischemia in the absence of significant epicardial coronary obstruction, supporting the link between affective disorders and ischemia with non-obstructive coronary arteries [66,68]. This integrative framework also provides a pathophysiological basis for stress-related CV phenotypes, such as takotsubo syndrome, in which acute emotional or physical stress triggers transient myocardial dysfunction through catecholamine-mediated mechanisms and altered brain–heart signaling [101,102]. Behavioral and psychosocial factors are integral components of this axis. In women, gender-related exposures, including caregiving roles, interpersonal stress, and social expectations, may amplify this interplay, contributing to cumulative vulnerability across the life course [103]. Integrating this perspective into clinical practice may facilitate more precise risk stratification and support the development of multidimensional, women-centered prevention strategies that address both biological and psychosocial determinants of CV health [75].

### 7.2. Stress-Related Cardiovascular Phenotypes: Takotsubo as a Heart–Brain Model

A clinically paradigmatic example of the heart-brain-stress interface, highly relevant to women’s CV health, is takotsubo (stress) cardiomyopathy, a unique and heterogeneous syndrome characterized by transient ventricular dysfunction and variable morphologic patterns [104]. The clinical spectrum of takotsubo is broader than initially appreciated, encompassing diverse triggers, presentations, and patterns of ventricular involvement, with implications for diagnostic framing and follow-up [105,106]. Early imaging-oriented descriptions highlighted variable ventricular morphology, supporting the concept of a syndrome rather than a single uniform phenotype [105].

Subsequent observational studies expanded the clinical profile and natural history, documenting a wide range of acute presentations that can mimic acute coronary syndromes and require careful integration of clinical, imaging, and biomarker data [106]. Population-level analyses have also provided epidemiologic grounding by estimating prevalence in real-world settings, reinforcing its relevance as a non-negligible stress-related entity within contemporary CV care [107]. Importantly, recent work emphasizes that sex- and gender-related differences should inform a personalized approach to takotsubo management in women, integrating biological susceptibility, psychosocial exposures, and phenotype in risk stratification and longitudinal care planning [102].

Epidemiological observations consistently show a marked female predominance, reinforcing the concept that sex- and gender-linked vulnerability to psychosocial stressors may translate into distinct CV phenotypes [2,3,107]. Beyond the psychosocial trigger itself, mechanistic studies support a central role for sympathetic activation and catecholamine-mediated myocardial stunning, further corroborated by reports of stress cardiomyopathy occurring after exogenous catecholamine or beta-agonist exposure, also related to allergic trigger [108,109,110]. Importantly, emerging neurobiological evidence suggests that takotsubo is not merely a “cardiac” condition but reflects altered brain–heart network activation in susceptible individuals, aligning with broader models in which chronic stress, anxiety, and depression modulate CV risk through neuroendocrine and autonomic pathways [101,102]. Neuroimaging studies have identified structural and functional abnormalities in brain regions involved in emotional processing, autonomic regulation, and stress perception, including the amygdala, insula, hippocampus, anterior cingulate cortex, and prefrontal cortex [111,112,113]. Functional magnetic resonance imaging studies have demonstrated altered connectivity within central autonomic networks, suggesting that abnormal neural processing of emotional and physical stressors may contribute to exaggerated sympathetic responses in susceptible individuals [103]. Particularly intriguing is the observation that increased resting amygdalar activity may precede the development of stress-related cardiovascular events, supporting the hypothesis that chronic neural stress signaling contributes to cardiovascular vulnerability [114].

Although left ventricular systolic function usually recovers within weeks, contemporary evidence indicates that takotsubo syndrome should not be regarded as a benign condition. Data from the International Takotsubo Registry demonstrated rates of death and major adverse cardiovascular and cerebrovascular events comparable to those observed in patients with acute coronary syndromes [103]. Furthermore, recurrent episodes occur in a clinically relevant proportion of patients, and many individuals continue to experience persistent symptoms, anxiety, depressive symptoms, and impaired quality of life despite apparent recovery of ventricular function [104]. These observations suggest that psychological assessment should not be limited to the acute phase but should be incorporated into longitudinal follow-up. Systematic screening for anxiety, depression, chronic stress burden, and major psychosocial stressors may help identify vulnerable patients who could benefit from multidisciplinary management, psychological support, and stress-reduction interventions [115].

Recent sex- and gender-focused syntheses further emphasize that takotsubo should be interpreted through a personalized lens, integrating biological susceptibility, gender-related exposures, and clinical phenotype to guide risk stratification and follow-up in women. In this framework, takotsubo becomes a high-yield “model condition” illustrating how psychosocial stress and affective symptoms can act as amplifiers of CV vulnerability, with potential implications for tailored management pathways [102]. These observations have practical implications for prevention and care pathways in women. First, they support a shift from viewing psychosocial distress as an “adjunctive” issue to recognizing it as a clinically actionable risk modifier, particularly in women presenting with ischemia-like symptoms, microvascular angina phenotypes, or recurrent stress-related presentations, where comprehensive risk appraisal should integrate both biological sex and gender-related exposures [2,3]. Second, they strengthen the rationale for multidisciplinary models, women’s heart clinics and integrated cardio-psychology services, capable of identifying psychosocial triggers, screening for anxiety/depression, and implementing tailored interventions that may improve symptom burden, risk-factor control, and adherence [4].

## 8. Clinical Implications: Screening, Prevention and Management

The recognition of anxiety, depression, and psychosocial stress as clinically relevant cardiovascular risk modifiers in women raises an important question: Can treatment of mental health disorders improve cardiovascular outcomes? Although the available evidence does not yet support a definitive reduction in major adverse cardiovascular events (MACE) through mental health interventions alone, growing data suggest that effective management of anxiety and depression may improve quality of life, treatment adherence, lifestyle behaviors, and overall cardiovascular risk profiles [4,74,116].

Building on the life-course framework described above, the integration of psychosocial assessment into CV care requires structured and pragmatic clinical pathways. The recognition of anxiety, depression, and psychosocial stress as clinically relevant CV risk modifiers in women has important implications for screening, prevention, and long-term management [63]. Translating this evidence into practice requires a structured, women-centered approach that integrates mental health assessment into CV care pathways.

**Figure 4:**
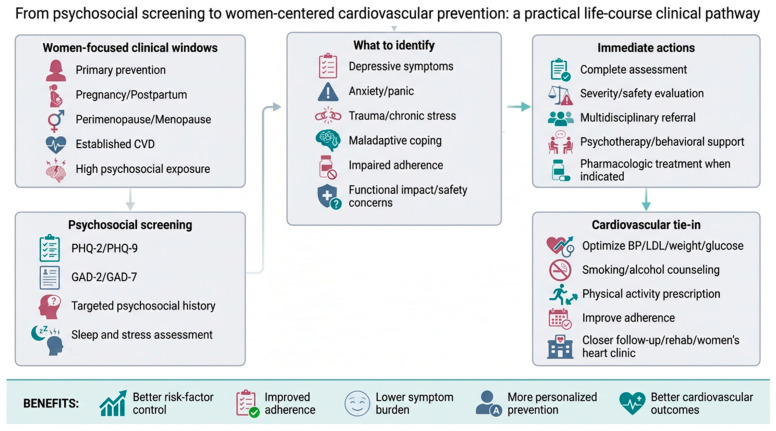
From psychosocial screening to women-centered cardiovascular prevention: a practical life-course clinical pathway. This figure outlines a pragmatic workflow for integrating anxiety and depression assessment into cardiovascular prevention in women across key life-course stages and clinical settings. Women-focused clinical windows include primary prevention, pregnancy and postpartum, perimenopause and menopause, established cardiovascular disease, and conditions characterized by high psychosocial exposure. Screening tools and targeted psychosocial history may help identify depressive symptoms, anxiety, trauma exposure, maladaptive coping, functional impairment, and barriers to adherence. These findings should prompt tailored multidisciplinary actions, including further assessment, safety evaluation, psychological or psychiatric referral when indicated, and implementation of prevention-oriented cardiovascular measures such as optimization of blood pressure, lipids, weight, glycemic control, smoking cessation, physical activity, rehabilitation, and closer longitudinal follow-up. The figure frames psychosocial distress as a clinically actionable cardiovascular risk modifier in women. Abbreviations: ASCVD, atherosclerotic cardiovascular disease; BP, blood pressure; CVD, cardiovascular disease; CV, cardiovascular; HbA1c, glycated hemoglobin; PTSD, post-traumatic stress disorder; PHQ-9, Patient Health Questionnaire-9; GAD-7, Generalized Anxiety Disorder-7.

Management should include both non-pharmacological and pharmacological approaches, framed within an integrated bio–psycho–social model of CV prevention in women [4,100]. A practical framework linking psychosocial screening to women-centered CV prevention actions across the life course is presented in Figure 4.

To facilitate clinical implementation, Table 2 proposes a pragmatic, women-centered screening-to-action pathway that links anxiety/depression assessment to tailored CV prevention strategies across different life-course stages and risk settings.

### 8.1. Screening and Identification of Affective Vulnerability

The first priority is the systematic identification of anxiety and depressive symptoms in women across different clinical settings. Brief, validated screening tools such as the Patient Health Questionnaire (PHQ-9) and the Generalized Anxiety Disorder scale (GAD-7) provide practical and scalable instruments for routine use in primary and cardiovascular care [71]. Screening should not be limited to psychiatric settings but incorporated into cardiovascular risk assessment, particularly in high-risk contexts such as primary prevention visits, pregnancy and postpartum care, the menopausal transition, and secondary prevention in patients with established cardiovascular disease [81,94]. In addition to standardized tools, targeted psychosocial history is essential to identify high-risk exposures, including interpersonal violence, chronic stress, and adverse life events, which may not be captured by symptom-based scales [34,36]. A trauma-informed approach is particularly important in women, given the high prevalence and cardiovascular relevance of these exposures.

### 8.2. From Screening to Action: Integrating Mental Health into Cardiovascular Prevention

Screening alone is insufficient unless it is linked to actionable clinical pathways. The identification of anxiety or depressive symptoms should prompt a graded response based on severity, functional impact, and clinical context. This may include further assessment, close follow-up, referral to mental health services, and, when appropriate, initiation of psychological or pharmacological treatment when clinically indicated [70,117].

Importantly, mental health assessment should be integrated with cardiovascular risk management. As discussed above, depressive and anxiety symptoms may adversely affect adherence and engagement with preventive care. Practical measures include simplifying treatment regimens, increasing follow-up frequency, and leveraging multidisciplinary care models [5]. Given the prognostic relevance of depressive symptoms in coronary disease and post-myocardial infarction settings [6,7,71], a pragmatic clinical priority is early identification of affective vulnerability and its translation into actionable prevention steps. In women, this requires a comprehensive appraisal that integrates traditional risk factors with sex- and gender-informed determinants, including psychosocial exposures that may shape symptom burden, healthcare engagement, and adherence to cardioprotective therapies [2,3].

### 8.3. Lifestyle, Behavioral, Psychological and Cardiac Rehabilitation Interventions

Non-pharmacological interventions represent a cornerstone of management. They should be positioned as core components of prevention-oriented care: structured education, lifestyle programs, and rehabilitation pathways (including CV prevention and, when relevant, cardiac rehabilitation) can provide a scalable infrastructure to address risk behaviors and improve longitudinal engagement [5]. Structured lifestyle interventions, including physical activity programs, dietary counseling, and smoking cessation support, are essential not only for cardiovascular risk reduction but also for improving mental health outcomes [70,118]. Exercise, in particular, has well-documented benefits on depressive symptoms, with meta-analytic data suggesting an extremely low number needed to treat of 2, while also exerting favorable effects on cardiometabolic risk [119,120,121,122].

Evidence-based psychological interventions may be integrated within multidisciplinary models, such as women’s heart clinics and integrated cardio-psychology services, to target distress-related symptom amplification, maladaptive coping and treatment discontinuation [5]. In parallel, the conceptual framework of personalized care, emphasized in sex- and gender-focused analyses of stress-related CV phenotypes such as takotsubo syndrome, supports tailoring follow-up intensity and care pathways to individual vulnerability profiles [102].

Cognitive behavioral therapy (CBT) is the most extensively studied psychological intervention and has consistently demonstrated efficacy in reducing depressive and anxiety symptoms across a broad range of patient populations [123]. Stress-management programs, mindfulness-based interventions, and structured psychosocial support may also improve psychological well-being, perceived stress, and health-related quality of life [124].

Importantly, treatment of depression and anxiety may confer cardiovascular benefits beyond symptom control. Mental health disorders are associated with lower adherence to preventive therapies, reduced participation in cardiac rehabilitation, unhealthy lifestyle behaviors, and delayed healthcare seeking. Consequently, successful treatment may indirectly improve cardiovascular prognosis by enhancing medication adherence, promoting healthier behaviors, and increasing engagement with healthcare services [4,71,74]. Nevertheless, evidence supporting a direct reduction in cardiovascular events remains heterogeneous. Large trials such as ENRICHD demonstrated significant improvements in depression and social support after myocardial infarction but did not show a clear reduction in mortality or recurrent cardiovascular events [125]. Similarly, subsequent studies and meta-analyses have generally reported consistent improvements in psychological outcomes and quality of life, whereas effects on hard cardiovascular endpoints have been less robust and often statistically inconclusive [126,127].

Cardiac rehabilitation programs provide an ideal platform for integrating psychological support with cardiovascular prevention, as psychosocial assessment and intervention are core components of comprehensive rehabilitation. In women, however, participation remains suboptimal, and depressive symptoms may further reduce program completion and engagement [128,129]. Accordingly, targeted strategies, including tailored communication, flexible or home-based program models, and structured psychosocial support, are crucial to improve uptake and adherence [130,131].

### 8.4. Pharmacological Considerations

Pharmacological treatment of anxiety and depression may be indicated in selected patients and should be individualized, taking into account cardiovascular comorbidities, potential drug interactions, and sex-specific factors [132,133,134,135,136]. In women with CV disease, particular attention should be paid to polypharmacy, autonomic symptoms, and potential pro-arrhythmic effects, especially in the context of QT interval modulation or arrhythmic vulnerability [5]. From a pharmacological perspective, selective serotonin reuptake inhibitors (SSRIs), including sertraline, escitalopram, fluoxetine, and paroxetine, are generally considered the preferred antidepressant agents in patients with established cardiovascular disease because of their favorable cardiovascular safety profile [116,137].

Among these, sertraline has been the most extensively studied in patients with coronary artery disease and post-myocardial infarction depression. In the SADHART trial, sertraline was shown to be safe and well tolerated in patients with recent acute coronary syndromes and major depression, without adverse effects on cardiac function, arrhythmias, blood pressure, or left ventricular ejection fraction [138]. Serotonin–norepinephrine reuptake inhibitors (SNRIs), including venlafaxine and duloxetine, are generally considered acceptable alternatives; however, caution may be warranted in patients with poorly controlled hypertension or significant cardiovascular disease because of their potential to increase heart rate and blood pressure [139]. By contrast, tricyclic antidepressants (TCAs), such as amitriptyline, nortriptyline, and imipramine, are generally avoided in patients with significant cardiovascular disease because of their proarrhythmic potential, effects on cardiac conduction, orthostatic hypotension, and risk of QT prolongation [137,140].

Particular caution is also recommended with high-dose citalopram because of its dose-dependent association with QT interval prolongation [141].

Beyond symptom control, the goal of pharmacological treatment in this context is to facilitate sustained participation in CV prevention strategies and to improve adherence to evidence-based therapies. This reinforces the concept of mental health disorders as modifiable CV risk enhancers, rather than merely isolated comorbidities [2,3,5]. Pharmacological and non-pharmacological interventions differ substantially in their cardiovascular safety profiles, mechanisms of action, and potential impact on adherence, lifestyle behaviors, and overall cardiovascular health. Table 3 summarizes the principal mental health interventions currently used in clinical practice and highlights their potential cardiovascular implications in women with established or at-risk cardiovascular disease [132,133,134,142,143,144].

### 8.5. Longitudinal and Life-Course-Oriented Care Models

Given the dynamic nature of psychosocial and biological vulnerability in women, cardiovascular prevention should adopt a longitudinal, life-course-oriented approach. Key windows, including pregnancy and postpartum, the menopausal transition, and periods of high psychosocial stress, represent opportunities for targeted screening and intervention [94,145]. Multidisciplinary models, such as women’s heart clinics and integrated cardio-psychology services, may provide an effective framework for addressing the complex interplay between mental health and cardiovascular risk [146]. These models allow for coordinated management of biological, behavioral, and psychosocial determinants, improving risk-factor control, symptom burden, and patient engagement [4,117,147]. In this framework, mental health should be considered not as an ancillary comorbidity but as a clinically actionable cardiovascular risk modifier, requiring integration into routine prevention strategies and longitudinal care pathways in women [70,118].

## 9. Conclusions

Cardiovascular disease in women cannot be fully understood through traditional risk-factor models alone [1,2,3,4]. Current evidence supports the treatment of anxiety and depression primarily to improve psychological health, functional status, and adherence to cardiovascular prevention strategies, while a potential direct impact on cardiovascular event reduction remains an area of ongoing investigation. In clinical practice, the management of women with cardiovascular disease should therefore adopt an integrated multidisciplinary approach in which mental health assessment and treatment are considered an integral component of cardiovascular prevention rather than a separate therapeutic domain [5]. The integration of sex-related biological mechanisms and gender-related exposures reveals a complex interplay between psychological, neuroendocrine, and vascular processes that shape cardiovascular vulnerability across the life course.

Anxiety, depression, and chronic psychosocial stress emerge not merely as comorbid conditions but as active modulators of cardiovascular risk in women, operating through interconnected pathways involving autonomic dysregulation, inflammation, endothelial dysfunction, and behavioral influences. These mechanisms are further amplified during specific female vulnerability windows, including reproductive transitions and periods of heightened psychosocial burden. The concept of a heart–brain–vascular axis provides a unifying framework to interpret these interactions, linking affective disorders and stress-related exposures to both chronic cardiovascular risk and acute clinical phenotypes, such as stress-induced myocardial ischemia and takotsubo syndrome. From a clinical perspective, these insights support a shift toward a more comprehensive, women-centered approach to cardiovascular prevention and care. Systematic screening for anxiety and depression, integration of psychosocial assessment into cardiovascular risk evaluation, and the implementation of multidisciplinary and life-course-oriented care models are essential steps toward improving outcomes.

Despite substantial advances in understanding the relationship between mental health and cardiovascular disease in women, important knowledge gaps remain. Future research should better clarify the causal pathways linking anxiety, depression, and psychosocial stress to cardiovascular outcomes, identify the biological and psychosocial determinants of individual susceptibility, and determine whether targeted mental health interventions can translate into measurable reductions in cardiovascular events. Additional priorities include the development of sex-specific risk prediction models incorporating psychosocial variables, validation of integrated screening strategies across different female life stages, and a deeper characterization of the heart–brain–vascular axis through multimodal imaging, biomarker, and longitudinal outcome studies. Addressing these gaps will be essential to move from association-based evidence toward precision prevention and personalized cardiovascular care for women.

Ultimately, recognizing mental health as a clinically actionable cardiovascular risk modifier may enable more precise risk stratification and more effective prevention strategies, contributing to a paradigm shift toward truly personalized, sex- and gender-informed cardiovascular medicine.

## Figures and Tables

**Table 1:** Mechanistic domains linking anxiety and depression to cardiovascular risk in women. Integrated biological and behavioral mechanisms through which anxiety and depressive disorders may contribute to cardiovascular risk and adverse cardiovascular outcomes in women. Level of evidence reflects the overall consistency of available epidemiological, mechanistic, and clinical data supporting each domain. “Strong” denotes mechanisms supported by robust and consistent evidence across multiple study designs, whereas “Moderate” denotes mechanisms supported by substantial but less definitive evidence. Abbreviations: BP, blood pressure; CV, cardiovascular; HbA1c, glycated hemoglobin; HPA, hypothalamic–pituitary–adrenal; LDL, low-density lipoprotein cholesterol.

Domain	Putative Mechanism(s)	Downstream CV Effects (Conceptual)	Clinical “Handles” (What Clinicians Can Act on)	Level of Evidence
Neuroendocrine stress systems	HPA axis dysregulation; stress-mediated hormonal perturbations	BP variability, metabolic dysregulation, pro-thrombotic milieu	Sleep optimization, stress reduction programs, structured follow-up	Moderate–Strong
Inflammation/immune activation	Chronic low-grade inflammation	Endothelial dysfunction; accelerated atherogenesis	Weight management, exercise prescription, control of comorbidities	Moderate
Autonomic imbalance	Sympathetic predominance; reduced vagal tone	Arrhythmia vulnerability; ischemia threshold changes	Physical activity, breathing/relaxation training, careful medication selection in arrhythmia-prone patients	Strong
Platelet/vascular function	Platelet activation; endothelial dysfunction	Thrombotic risk; microvascular dysfunction	Aggressive management of traditional risk factors; consider microvascular angina work-up when appropriate	Moderate
Behavioral pathways	Smoking, poor diet, inactivity, substance use	Worsened cardiometabolic profile	Brief interventions, structured lifestyle programs, referral to rehabilitation	Strong
Health system/adherence	Reduced adherence, low engagement, delayed care seeking	Suboptimal LDL/BP/HbA1c control; missed rehabilitation	Simplify regimens, frequent follow-up, digital adherence support	Strong

**Table 2:** The table proposes a pragmatic framework linking mental health screening to tailored cardiovascular prevention and management strategies across key female life stages and clinical settings. The approach emphasizes integration of psychosocial assessment into routine cardiovascular care, recognizing anxiety and depression as clinically relevant modifiers of cardiovascular risk and treatment adherence. Abbreviations: BP, blood pressure; CV, cardiovascular; GAD-2, Generalized Anxiety Disorder-2 questionnaire; GAD-7, Generalized Anxiety Disorder-7 questionnaire; HADS, Hospital Anxiety and Depression Scale; HF, heart failure; LDL, low-density lipoprotein cholesterol; PHQ-2, Patient Health Questionnaire-2; PHQ-9, Patient Health Questionnaire-9; QT, QT interval.

Setting/Timing	Screening Tool(s)	Key Findings to Identify	Immediate Actions	Cardiovascular Implications/Actions
Primary prevention visit (any age)	PHQ-2/PHQ-9; GAD-2/GAD-7	Persistent low mood, anhedonia, excessive worry, avoidance behaviors, sleep disturbance	If screening is positive, complete the full assessment scale; evaluate functional impairment and safety in patients with severe symptoms	Intensify lifestyle counseling; prioritize adherence to BP, LDL-C, and weight targets; address smoking and alcohol consumption; consider closer follow-up
Pregnancy/postpartum (cardio-obstetrics)	PHQ-9; GAD-7 (plus postpartum-specific screening according to local pathways)	New-onset depressive symptoms, panic symptoms, insomnia, intrusive worry, high psychosocial stress	Coordinate care between obstetrics, primary care, and mental health services; consider prompt referral for moderate-to-severe symptoms	Reinforce postpartum cardiometabolic surveillance (BP, weight, glucose, lipids); encourage gradual resumption of physical activity
Perimenopause/menopause transition	PHQ-9; GAD-7	Worsening mood or anxiety, vasomotor symptoms, sleep disturbance, weight gain	Perform a holistic assessment; consider behavioral interventions and/or psychotherapy; evaluate medication tolerability and QT-related issues when relevant	Reassess overall CV risk; optimize BP- and lipid-lowering therapies; address sleep and physical inactivity as risk mediators
Established cardiovascular disease (secondary prevention)	PHQ-9; GAD-7; HADS (where locally used)	Anxiety or depressive symptoms affecting rehabilitation attendance, treatment adherence, or self-care	Integrate screening within cardiac rehabilitation programs; consider psychotherapy and pharmacological treatment when indicated; monitor treatment-related adverse effects	Focus on adherence to preventive therapies (antiplatelets, statins, HF therapies); monitor arrhythmic risk and QT interval when appropriate; track achievement of risk-factor targets
High psychosocial exposure (violence, bullying, major life events)	Standard screening tools plus targeted psychosocial history	Trauma exposure, chronic stress, maladaptive coping strategies (smoking, binge eating, substance misuse)	Implement trauma-informed referral pathways; facilitate access to social support and mental health resources	Consider psychosocial burden as a CV risk enhancer; adopt tighter surveillance and more frequent preventive follow-up visits

**Table 3:** Practical cardiovascular considerations for mental health interventions in women with cardiovascular disease. Summary of the main pharmacological and non-pharmacological interventions used for anxiety and depression, with a focus on cardiovascular safety, potential cardiovascular benefits, and practical clinical considerations. Although improvement in psychological symptoms is consistently demonstrated, evidence for a direct reduction in major adverse cardiovascular events remains limited and heterogeneous. Clinical note: Selection of antidepressant therapy should be individualized according to psychiatric indication, cardiovascular comorbidities, arrhythmic risk, concomitant medications, and patient preferences, ideally within a multidisciplinary cardiovascular–mental health care pathway. Abbreviations: CBT, cognitive behavioral therapy; CV, cardiovascular; HR, heart rate; QT, electrocardiographic QT interval; SNRI, serotonin–norepinephrine reuptake inhibitor; SSRI, selective serotonin reuptake inhibitor; TCA, tricyclic antidepressant.

Intervention	Representative Agents/Interventions	Cardiovascular Safety Profile	Potential Cardiovascular Benefits	Practical Considerations
SSRIs	Sertraline, escitalopram, fluoxetine, paroxetine	Generally favorable	Improved adherence, symptom control, healthier lifestyle behaviors	Often considered first-line antidepressants in patients with cardiovascular disease
SNRIs	Venlafaxine, duloxetine	Generally acceptable	Improvement of anxiety and depressive symptoms	Monitor blood pressure and heart rate, particularly in hypertensive patients
TCAs	Amitriptyline, nortriptyline, imipramine	Less favorable	Effective antidepressant activity in selected patients	Potential QT prolongation, conduction abnormalities, orthostatic hypotension, and arrhythmias
Cognitive behavioral therapy (CBT)	Structured psychotherapy	Excellent	Reduced depression and anxiety burden, improved adherence and quality of life	Recommended as a core component of multidisciplinary care
Stress-management and mindfulness interventions	Behavioral and stress-reduction programs	Excellent	Improved psychological well-being and stress perception	May be integrated into preventive cardiovascular programs and cardiac rehabilitation
Multidisciplinary care	Cardiology–mental health collaboration	Excellent	Better risk-factor control, adherence, and patient engagement	Particularly relevant in women with high psychosocial burden

## Data Availability

No new data were created or analyzed in this study. Data sharing is not applicable to this article.

## Abbreviations

The following abbreviations are used in this manuscript:

ACS	acute coronary syndromes
BP	blood pressure
CV	cardiovascular
CVD	cardiovascular disease
GAD-2	2-item Generalized Anxiety Disorder scale
GAD-7	7-item Generalized Anxiety Disorder scale
HbA1c	glycated hemoglobin
HADS	Hospital Anxiety and Depression Scale
HDL	high-density lipoprotein
HPA	hypothalamic–pituitary–adrenal
INOCA	ischemia with non-obstructive coronary arteries
LDL	low-density lipoprotein
PHQ-2	2-item Patient Health Questionnaire
PHQ-9	9-item Patient Health Questionnaire
PMDD	premenstrual dysphoric disorder
PTSD	post-traumatic stress disorder
QT	QT interval
